# 

**Published:** 1905-01

**Authors:** 


### LIST OF CONTRIBUTORS TO VOLUME XXVI.

---

Allan, Charles F., M.D.
Angle, Edward H., M.D., D.D.S.
Baker, Lawrence W., D.M.D.
Boenning, Henry C., M.D.
Bogue, E. A., M.D., D.D.S.
Brigham, Dr. Walter I.
Brown, Geo. V.I., A.B., D.D.S., M.D., C.M
Brush, Dr. F. C.
Byram, Dr. J. Q.
Carlton, Harry P., D.D.S.
Chittenden, Charles C., D.D.S.
Crothers, T. D., M.D.
Cryer, M. H., M.D., D.D.S.
Davenport, Dr. S. E.
Dowski, Samuel, D.D.S.
Fillebrown, Thomas, M.D. D.M.D.
Fossume, Dr. F. L.
Gelston, William H., D.D.S.
Goslee, Hart J., D.D.S.
Hamilton, Dr. Harry F.
Howe, Horace L., D.M.D.
Hofheinz, R. H., D.D.S.
Hopkins, Samuel A., M.D., D.D.S.
Hurd, Lee Maidmont, M.D.
Jenkins, Dr. N. S.
Lawshe, Allison, R., D.D.S.
Lederer, Wm. J., D.D.S.
Leonard, T. M. Russell, L.R.C.P., L.R.C. S. (Ed.).
Lewis, O. G. L., D.D.S.
MacPherson, Dr. A. H.
McLaren, F. J.
Miller, Willoughby D., A.M., M.D., D.D.S., Sc.D.
McManus, Dr. Charles.
O'Brian, Dr. L. A.
Palmer, James G.
Rhein, M. L., M.D., D.D.S.
Richards, George L., M.D.
Rollins, William.
Schamberg, M. I., D.D.S., M.D.
Smith, H. Carleton, Ph.D.
Spaulding, John H., D.D.S.
Stern, Heinrich, M.D.
Strang, Dr. C. W.
Trueman, William H., D.D.S.
Talbot, Eugene S., M.S., D.D.S., M.D., LL.D.
Truman, James, D.D.S., LL.D.
Wedelstaedt, E. K., D.D.S.
Wheeler, Herbert Locke, D.D.S.
Whitlock, Dr. W. M.
Wright, C. M., A.M., D.D.S.

